# Multiple Muscle Metastases as the First Presentation of Gastric Cancer: A Case Report and Review of Literature

**DOI:** 10.7759/cureus.55458

**Published:** 2024-03-03

**Authors:** Polyxeni Pichioni, Dimitrios Kokkinovasilis, Stylianos Stylianou, Georgios Kipouridis, Alkiviadis Kalogeropoulos, Saant Al Mogrampi

**Affiliations:** 1 Department of Surgery, General Hospital of Imathia, Naousa Health Unit, Naousa, GRC

**Keywords:** muscle metastasis, gastric metastasis, abdominal wall mass, abdominal wall invasion, gastric cancer

## Abstract

The presence of an abdominal wall mass may serve as the initial presentation of an unknown gastric malignancy. The invasion of the abdominal wall and the occurrence of multiple skeletal muscle metastases originating from gastric cancer are exceedingly uncommon. We present a case of a 45-year-old female patient exhibiting widespread abdominal wall infiltration and skeletal muscle metastases derived from gastric cancer. The primary presentation included a distressing diffuse abdominal mass in the left upper and lower quadrants. Abdominal computed tomography revealed extensive swelling of multiple skeletal muscles within the abdominal wall, raising suspicions of gastric malignancy. Biopsies of the affected muscles, along with upper gastrointestinal tract endoscopy and colonoscopy, were performed. The upper endoscopy examination unveiled a poorly differentiated diffuse-type gastric adenocarcinoma, while the subsequent muscle biopsy confirmed infiltration by the recently diagnosed malignancy. At this stage of the disease, systemic chemotherapy was deemed the optimal choice for our patient. Subsequent abdominal computed tomography showed a decrease in the dimensions of the abdominal wall and other skeletal muscle lesions. Seventeen months after the initial diagnosis, our patient continues to be alive. Additionally, we provide a comprehensive review of the existing literature on similar reported cases of gastric cancer patients with concurrent muscle metastases.

## Introduction

Gastric cancer ranks as the fifth most prevalent malignancy and stands as the third most frequent cause of cancer-associated mortality globally [1]. The vast majority of malignant gastric tumors are adenocarcinomas [2]. According to the Lauren classification system, gastric adenocarcinomas can be histologically classified into two subgroups: diffuse type and intestinal type. The intestinal type of gastric carcinomas is usually linked to lymphatic or hematogenous metastases. Therefore, lesions in distant areas of the body are common in this type. It primarily occurs in older male patients and has a better prognosis. On the contrary, the diffuse type usually affects younger female patients and has a worse prognosis compared to the intestinal type. Peritoneal dissemination is usual in diffuse gastric carcinomas [3]. The prognosis of patients diagnosed with gastric carcinoma hinges upon the cancer stage, ascertained by the extent of tumor invasion, lymph node involvement, and the presence or absence of metastases [4]. Gastric cancer can disseminate through multiple pathways, encompassing lymphatic and hematogenous spread, subperitoneal dissemination, direct invasion into neighboring organs, and peritoneal cavity dissemination [5]. Gastric cancer can be categorized into four stages (I-IV) according to the Japanese Classification of gastric carcinoma. Having a metastasis to areas of the body other than the regional lymph nodes or adjacent anatomical structures is classified as a distant metastasis. In such cases, the patient is considered to have stage IV gastric carcinoma [6]. The literature has scarcely reported cases of abdominal wall invasion from gastric cancer, as well as muscle metastases arising from gastric malignancy. We present a case report of a female patient exhibiting diffuse abdominal wall swelling, diagnosed as abdominal wall invasion and concurrent multiple muscle metastases originating from gastric cancer.

## Case presentation

On September 20, 2022, a 45-year-old female was admitted to the surgical department of our hospital due to a painful left abdominal wall mass. The patient reported experiencing fatigue and weight loss over the previous five months. Vitals and laboratory test results were generally within normal ranges, except for a low hematocrit level (32.0%), which the patient had disclosed during admission. A thorough clinical examination revealed a sizable and tender mass in the left upper and lower quadrants of the abdomen.

**Figure 1:**
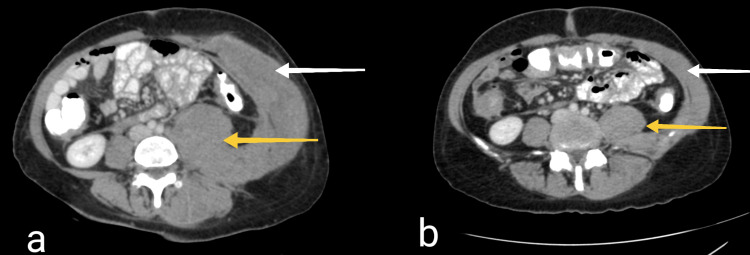
Contrast-enhanced abdominal computed tomography (a) The initially performed CT demonstrates diffuse thickening of the gastric wall, epiploic seeding, multisegmental serosal thickening of the gastrointestinal tract, as well as dramatic diffuse thickening of the muscles of the back (yellow arrow) and the anterolateral abdominal wall on the left side (white arrow). (b) The CT performed 16 months after the initial diagnosis, after the completion of chemotherapy, demonstrates a substantial improvement concerning primarily the abdominal wall invasion.

A contrast-enhanced computed tomography (CT) scan of the abdomen revealed extensive swelling of multiple skeletal muscles of the back and the anterolateral abdominal wall on the left side, as well as thickening of the gastric wall and the serosa layer of the gastrointestinal tract, prompting suspicion of gastric malignancy (Figure 1a). A biopsy of the affected abdominal wall muscles and an upper gastrointestinal tract endoscopy and colonoscopy were performed. The gastroscopy unveiled a 25-mm diameter ulcer located at the body of the stomach, and a subsequent biopsy of the ulcer confirmed the existence of a poorly differentiated diffuse-type adenocarcinoma. The colonoscopy revealed no abnormalities. The muscle biopsy results demonstrated diffuse infiltration of the skeletal muscles by the recently diagnosed gastric adenocarcinoma. Additionally, the chest CT scan exhibited no pathological findings.

The patient was referred to the Oncology Department to commence treatment. Based on the CT findings, the biopsy results from the abdominal wall muscles, and the findings of the upper endoscopy, our patient received a diagnosis of stage IV gastric adenocarcinoma, according to the Japanese Classification of Gastric Carcinoma [6]. At this point, the patient's treatment plan was optimized through the selection of systemic chemotherapy as the most suitable course of action. Subsequent to chemotherapy, CT scans of the chest and abdomen demonstrated a reduction in the size of the skeletal muscle metastases and the lesions within the peritoneal cavity (Figure 1b). Seventeen months following the initial diagnosis, the patient has regained her normal weight, has returned to her previous job and is currently under surveillance and careful monitoring from her physicians.

## Discussion

Abdominal wall masses can manifest as the chief concern in patients seeking care at the emergency department or can be serendipitously identified through incidental imaging procedures. Research indicates that the predominant sources of abdominal wall masses include desmoid tumors, sarcomas, metastatic growths, lipomas, and endometriomas, with a substantial portion (approximately 58%) exhibiting benign characteristics [7]. When dealing with extensive abdominal wall conditions, such as the one presented in our scenario, the diagnostic strategy necessitates a comprehensive understanding of the patient's medical history [8]. In their investigation, Li et al. delineated a diagnostic methodology for discerning abdominal wall masses by categorizing them into three distinct classes: primary neoplasms, secondary malignancies, and neoplasm-mimicking lesions [9]. Primary neoplasms encompass entities like lipomas, liposarcomas, fibroblastic and vascular tumors, nerve sheath tumors, and undifferentiated sarcomas. Secondary neoplasms relate to instances of metastases and lymphomas. Abdominal wall infiltration by secondary growths occurs through mechanisms of metastasis, direct encroachment, and implantation from remote locations. Additionally, tumor-like formations include hernias, endometriosis, hematoma, and abscesses. According to the guidelines established by the American College of Radiology (ACR) Appropriateness Criteria for evaluating abdominal wall masses, diagnostic modalities such as ultrasound, contrast-enhanced CT, and MRI are deemed suitable techniques for visualization [10].

The invasion of the abdominal wall by gastric cancer in patients is an exceedingly uncommon occurrence. As far as current understanding extends, only a handful of instances have been documented in the available literature (Table 1). The prevailing trend in the reported cases involves the implementation of an aggressive surgical strategy, characterized by comprehensive gastrectomy and simultaneous en bloc removal of the infiltrated adjacent organs.

**Table 1:** Reported cases of abdominal wall invasion in patients with gastric cancer [11-17]

Authors	Year	Age	Sex	Other sites of metastasis	Treatment	Survival
Chu et al. [11]	1995	61	Female	Many adjacent organs	Gastrectomy, resection of affected organs	6 months (alive)
Katsumoto et al. [12]	2007	68	Female	Abdominal tumors	Distal gastrectomy, systemic chemotherapy	Not Available
Carlomagno et al. [13]	2015	81	Female	Transverse mesocolon	Gastrectomy, transverse colon and abdominal wall resection	Died 20 days post-operation
Cho et al. [14]	2015	71	Female	None	Gastrectomy, partial resection of abdominal wall	Died 3 months post-operation
Misumi et al. [15]	2019	76	Male	None	Distal gastrectomy, repeated extractions of abdominal wall masses	1 year and 6 months post operation (alive)
Nakamura et al. [16]	2021	70	Male	Transverse colon	Gastrectomy, transverse colon and abdominal wall resection, chemotherapy	6 months post operation (alive)
Fukui et al. [17]	2021	75	Male	Port site metastasis, left gluteal subcutaneous metastasis	Laparoscopic total gastrectomy, mass resection, radiotherapy	78 months post gastrectomy (alive)
This study	2024	45	Female	Multiple muscle metastases, peritoneal dissemination	Systemic chemotherapy	17 months (alive)

McNeer et al. emerged as early contributors to the notion that individuals afflicted with locally advanced gastric cancer necessitate interventions beyond gastrectomy to optimize their life expectancy [18]. A study encompassing 281 patients diagnosed with gastric cancer coupled with invasions into adjacent anatomical structures unveiled that survival rates could be extended by excising not only the stomach but also the invaded organs. This survival benefit was contingent upon the absence of peritoneal cavity dissemination or extensive nodal engagement [19]. In contrast to these typical scenarios, our presented case diverges due to the infiltration of numerous muscles by the primary gastric carcinoma. While the norm features anterior abdominal wall involvement, primarily concerning the rectus abdominis muscle, our instance deviates as it encompasses not only abdominal wall muscles but also back muscles within the patient's widespread swelling. This unique presentation led to the diagnosis of multiple muscle metastases.

The occurrence of skeletal muscle metastasis has been ascertained to be approximately 0.03% to 0.16% [20]. It remains challenging to elucidate the rarity of metastasis to muscle, especially considering that skeletal muscles constitute more than half of the body's total mass, as noted by Oba et al. [21]. The authors proposed several factors contributing to this rarity, including the continuous blood circulation, the potential clearance of malignant cells through muscle contractions, and the impediments posed to tumor cell proliferation by factors such as lactic acid, proteases, and muscle pH.

Haygood et al. conducted a comprehensive review encompassing 264 cases of patients afflicted with skeletal muscle metastases [22]. Their analysis divulged that the majority of these cases originated in the lungs, with only one instance stemming from the stomach as the primary source. In a separate study, Herring et al. chronicled 15 patients who exhibited skeletal muscle metastases over a 16-year period, alongside an examination of 52 additional cases documented in the literature [23]. Within their cohort, a solitary patient displayed a gastrointestinal tract primary tumor, while once again, 53% of cases were traced back to pulmonary origins. Upon scrutinizing the literature, Herring et al. discovered that 23% of all cases featuring skeletal muscle metastases had their origins in the gastrointestinal tract, and notably, only five out of the 52 cases featured multiple muscle metastatic lesions.

Despite CT scans often being the initial means of identifying muscle metastases, MRI stands out as a more adept modality for detecting these anomalies. Upon recognizing a mass within skeletal muscle, biopsy becomes imperative for accurate diagnosis [23].

Since 2000, a total of 22 instances involving muscle metastases originating from gastric cancer have been documented (Table 2). It remains plausible for muscle metastases to serve as the primary clinical indication of an occult primary lesion, either with or without concurrent metastases elsewhere. In individuals with a known malignancy history, the emergence of a painful mass should prompt clinicians' suspicion, with the patient's medical background pivotal in the final diagnosis determination [20].

**Table 2:** Reported cases of gastric cancer patients with concurrent muscle metastases [20-21, 24-42]

Authors	Year	Age	Sex	Sites of muscle metastases	Other sites of metastases	Treatment for gastric cancer	Treatment for muscle metastases	Survival after the muscle metastasis diagnosis
Oba et al. [21]	2001	70	Male	Left lumbar muscle, left iliopsoas muscle	Brain, liver, lungs, adrenal glands	None	None	71 days
Kondo et al. [24]	2002	64	Female	Left gluteus maximus, left adductor magnus muscle	Abdominal wall	Total gastrectomy with splenectomy	Tumor excision of left gluteal mass, chemotherapy	13 months
Tuoheti et al. [20]	2004	48	Male	Gluteal muscle	Not available	Excision	Wide excision	6 months
Tuoheti et al. [20]	2004	89	Male	Shoulder muscle	Not available	Excision	Radiation therapy	10 months (alive)
Bese et al. [25]	2006	60	Male	Paravertebral muscle	Perigastric and lumboaortic lymph nodes	Gastrectomy, chemoradiotherapy	Palliative chemoradiotherapy	Not available
Souayah et al. [26]	2008	49	Male	Right lateral rectus muscle	Not Available	Radiotherapy	Radiotherapy	10 weeks
Tougeron et al. [27]	2009	71	Male	Deltoid muscle	None	Partial gastrectomy, chemoradiotherapy	Chemoradiotherapy	13 months (alive)
Satonaka et al. [28]	2010	51	Male	Right thigh	Lungs, brain, skin	Chemotherapy, radiotherapy	Chemotherapy, radiotherapy	7 months
Sakuma et al. [29]	2011	64	Female	Gluteal muscle	Retroperitoneal and peritoneal dissemination	Total gastrectomy	Chemotherapy	18 months (alive)
Gogou et al. [30]	2012	Not available	Male	Femoral muscle	No liver or lung metastases	Gastrectomy	Wide excision, radiotherapy	30 months
Pergolini et al. [31]	2014	67	Male	Adductor muscle	Widespread metastatic disease	Chemotherapy	Chemotherapy	74 days
Lourenço et al. [32]	2014	68	Male	Right thigh	None	Chemotherapy	Chemotherapy	Not available
Koga et al. [33]	2015	71	Male	Latissimus dorsi, transverse abdominal, iliopsoas, femoral muscle	None	Preoperative chemotherapy, total gastrectomy	Chemotherapy	18 days
Temido et al. [34]	2017	42	Male	Extraocular muscle	Bone metastasis, mediastinal and abdominal ganglia	None	None	Shortly after diagnosis
Kamitani et al. [35]	2018	47	Male	Left latissimus dorsi, paraspinal muscle, quadriceps	None	Distal gastrectomy, chemotherapy	Chemotherapy	7 months
Aguirre et al. [36]	2019	57	Female	Obturator internus, vastus lateralis, quadratus lumborum, psoas, gluteus maximus, piriformis muscle	Peritoneal carcinomatosis	Total gastrectomy, chemoradiation	Palliative radiotherapy	3 months after discharge
Goto et al. [37]	2019	54	Female	Left medial rectus muscle	Ovary and mesentery, thoracic bone marrow	Chemotherapy	Radiotherapy	3 months
Korehisa et al. [38]	2021	64	Male	Right gluteal muscle	Peritoneal dissemination	Distal gastrectomy, chemotherapy	Chemotherapy, radiotherapy	2 months
Garcia et al. [39]	2021	44	Female	Left deltoid muscle	None	Gastrectomy, chemotherapy	Excision	Not available
Daneti et al. [40]	2021	42	Male	Multiple muscle metastases	Not available	Antropyloric stenting	Supportive care	6 weeks
Sellami et al. [41]	2022	53	Male	Right superior oblique muscle	Lungs	Chemotherapy	Chemotherapy	Died before completing his courses of chemotherapy
Roohe et al. [42]	2022	67	Male	Left rectus lateralis muscle	Leptomeningeal metastasis	None	None	2 months
Our case	2024	45	Female	Multiple muscle metastases	Peritoneal dissemination	Chemotherapy	Chemotherapy	17 months (alive)

Potential therapeutic avenues encompass radiotherapy, chemotherapy, or surgical excision, contingent on the clinical attributes of the muscle metastasis. For patients bearing exclusively a painful muscle metastasis without additional metastatic foci, judicious surgical removal might mitigate pain and extend life expectancy [23].

Our patient received a diagnosis of stage IV gastric cancer owing to the substantial invasion of skeletal muscles and the peritoneal dissemination of cancer cells. For individuals grappling with advanced gastric cancer or those who undergo non-curative R2 resection, systemic chemotherapy stands as the preferred therapeutic approach [43]. The anticipated survival period for patients confronting this stage of gastric cancer approximates 15 months [44]. Given the advanced stage in our case, radiotherapy and surgical excision were ruled out as feasible interventions. Our patient successfully underwent multiple rounds of chemotherapy, and as of 17 months post-diagnosis, she is presently alive and under careful monitoring.

## Conclusions

Abdominal wall invasion and muscle metastases from gastric cancer are rare and difficult to diagnose. Any muscle mass should raise suspicion to clinicians and lead to a biopsy in order to establish a diagnosis. Muscle metastases may be the first presentation of an unknown malignancy, as in our case.
